# Benefits of using the Brief Pain Inventory in patients with cancer pain: an intervention study conducted in Swedish hospitals

**DOI:** 10.1007/s00520-019-05200-6

**Published:** 2019-12-10

**Authors:** Viveka Andersson, Stefan Bergman, Ingela Henoch, Hanna Simonsson, Karin Ahlberg

**Affiliations:** 1 The Sahlgrenska Academy, Institute of Health and Care Sciences, University of Gothenburg, Box 457, SE-405 30 Gothenburg, Sweden; 2 Department of Medicine, Hallands Hospital Varberg, Träslövsvägen 68, 432 37 Varberg, Sweden; 3 Primary Health Care Unit, Department of Public Health and Community Medicine, Institute of Medicine, The Sahlgrenska Academy, University of Gothenburg, Box 457, SE-405 30 Gothenburg, Sweden; 4 Spenshult Research and Development Centre, Bäckagårdsvägen 47, 302 74 Halmstad, Sweden; 5 Angered Local Hospital, Halmtorget 1, 424 65 Gothenburg, Sweden; 6 Department of Surgery, Hallands Hospital Halmstad, Lasarettsvägen, 302 42 Halmstad, Sweden

**Keywords:** Cancer pain, Pain management, Pain prevalence, Hospital, Brief Pain Inventory, Edmonton Symptom Assessment Scale

## Abstract

**Purpose:**

The prevalence of cancer pain is too high. There is a need for improvement of pain management in cancer care. The aim of this study was to explore whether the use of the multidimensional pain assessment questionnaire Brief Pain Inventory (BPI) could improve pain relief in hospitalized patients with cancer.

**Methods:**

A controlled intervention study was performed at two hospitals in western Sweden, 264 patients were included, 132 formed a control group and 132 an intervention group. All participants completed the BPI and the Edmonton Symptom Assessment Scale (ESAS) at baseline. Only the researcher had access to questionnaires from the control group. The completed forms from the intervention group were presented to the patients’ care team. A follow-up took place after 2–5 days when patients in both groups rated the scales a second time.

**Results:**

In the intervention group, significant differences in all measured items of the BPI were found at follow-up compared with baseline. Symptoms rated with the ESAS also decreased significantly, except shortness of breath. At follow-up, a significant increase in regular use of paracetamol, anti-neuropathic pain drugs and opioids was found, as well as elevated doses of fixed-schedule opioids. In the control group, differences between baseline and follow-up were significant regarding average pain and worst pain over the past 24 h.

**Conclusion:**

Presenting the patient-reported BPI to the care team helped them to focus on patients’ pain, identify pain mechanisms and adjust analgesics accordingly. A possible explanation for the results is changes in the medication prescribed.

## Introduction

The prevalence of cancer pain is still far too high and has not improved significantly over the last decade [1], as compared to the four decades before [2]. A review article from 2016 reveals that pain occurs in 39% of patients after curative treatment, in 55% of those undergoing anticancer treatment and in 66% of those suffering from advanced cancer [3]. These figures are in line with those reported by the International Association for the Study of Pain (IASP) [4]. Despite increased focus in the field and greater knowledge of and possibilities for adequate pain relief, many patients are still affected by distressing or severe pain [5]. Pain impacts on quality of life (QoL), mood, sleep and the ability to perform activities of daily living [4, 6]. Barriers to adequate pain control have been identified, such as inadequate pain assessment, failure to use guidelines, reluctance to administer opioids, lack of knowledge, patients’ concerns about addiction and side effects as well as suboptimal education of healthcare professionals [7].

Pain in cancer patients is caused by the direct effect of the tumour or may be related to side effects of chemotherapy, radiation treatment and therapeutic procedures [8]. The most common pain mechanism is nociceptive pain, which occurs in 72% of those reporting pain, while 43% have elements of neuropathic pain and 30% have breakthrough pain [9]. Breakthrough pain is defined as an episode of severe pain that flares up during constant pain of mild to moderate intensity treated with an opioid [10]. Neuropathic pain is significantly more common among those undergoing oncological treatment, receiving strong opioids and having reduced performance status [11].

Evidence-based guidelines describing assessment and treatment of nociceptive, neuropathic and breakthrough pain are available [12, 13]. Teamwork in the treatment of cancer pain, comprising both pharmacological and non-pharmacological interventions, provides a wide range of options [13]. The need to alleviate pain and the availability of effective treatment regimens make it necessary for healthcare professionals to become skilled at assessing and treating cancer pain [13]. Guidelines also state that all patients should be screened for pain at each contact, and, where pain exists, a comprehensive pain assessment must be performed [12, 13].

The Brief Pain Inventory (BPI) is a patient-reported questionnaire, which is widely used for measuring pain intensity and how pain interferes with everyday life such as walking, general activity and work (activity dimension), sleep, mood, relations with others and enjoyment of life (affective dimension) [14–16]. There is a knowledge gap regarding potential effects on patient care and pain outcome when using the BPI.

The aim of this study was to explore whether the use of the multidimensional pain assessment questionnaire BPI could improve pain relief in hospitalized patients with cancer.

## Methods

### Design

A controlled intervention study was performed at a university hospital and a county hospital in western Sweden. A total of 264 patients were included.

### Participants

The patients included at the university hospital were recruited from the radiotherapy, oncology, haematology, pulmonary and palliative care units. At the county hospital, patients from the surgery, urology, medicine, pulmonary, head and neck and gynaecology units were included. The study was conducted over 4 months in 2016 and 4 months in 2017 at the county hospital and over 3 months in 2017 and 1 month in 2018 at the university hospital.

The inclusion criteria were inpatients who had been diagnosed with cancer, age ≥ 18 years, ability to understand and speak Swedish, a pain rating of moderate or higher (≥ 4) on a 0–10 numeric rating scale (NRS) and agreeing to participate in the study. Exclusion criteria were having undergone surgery during the previous 3 weeks, cognitive disability and critical illness.

#### Control group

The control group comprised the first half of the patients recruited from each hospital. They received their usual treatment.

#### Intervention group

The second half of patients at both hospitals constituted the intervention group. The reason was that we did not want staff to start using the BPI on all patients before the data collection was finished.

### Intervention

Participants in the intervention group completed the BPI multidimensional pain questionnaire and the Edmonton Symptom Assessment scale (ESAS) [17] at baseline. The completed questionnaires were presented to their respective care team and entered into their medical records as knowledge documentation. The researcher provided the care team with information about pain management guidelines when requested. The guidelines were provided in a pocket- sized booklet or on the intranet and comprised theoretical information about the physiology and dimensions of pain, as well as pharmacological and nonpharmacological treatment recommendations. Physicians then adjusted the analgesic dose based on patients’ reported BPI scores, descriptions of pain characteristics and the painful areas marked on the body chart.

Participants in the control group also completed the BPI and ESAS at baseline. Only the researchers had access to the control group questionnaires.

A follow-up took place after 2–5 days when patients in both the control group and the intervention group rated the BPI and ESAS a second time. Patients who had been discharged from hospital were contacted by phone. At follow**-**up the medical records were scrutinized for analgesic prescriptions.

### Outcomes

Demographic characteristics, i.e. age and sex, and clinical characteristics such as main diagnosis, documentation of pain and prescribed analgesic treatment were derived from the medical records. A structured, verbally administered questionnaire was used to obtain information from the patients, including the questions “In what year did you receive your diagnosis?”, “Have you undergone surgery? Radiation? Chemotherapy? Curative treatment?” and “Are you registered with a palliative consultation team or an advanced home care team?” If a patient was unable to answer a question, the nurse who cared for her/him was asked to provide the information.

Two additional questions were posed directly to the patient: “Have you previously used a pain rating scale during this hospital stay?” and “What do you consider effective for relieving pain?”

Pain during the previous 24 h was assessed using the BPI, which has been found to have good reliability and validity for measuring cancer pain [15, 18]. The BPI starts with an open question about the presence of pain. It includes a body chart to indicate areas of pain and the part of the body with the worst pain, with response alternatives for describing the characteristics of the pain. This is followed by four single-item measures of pain severity (present pain and least, average and worst pain). Each item is rated on an NRS from 0 = no pain to 10 = worst pain imaginable. One question concerns the patient’s current pain treatment. The pain relief experienced is indicated on a scale of 0–100%. The second part of the BPI assesses the extent to which pain interferes with seven aspects of function: general activity, mood, walking ability, normal work, relations with other people, sleep and enjoyment of life. Each item is rated on an NRS from 0 = no interference to 10 = interferes completely.

The ESAS was used to obtain information about other symptoms such as fatigue, nausea, downheartedness, tiredness, anxiety, drowsiness, loss of appetite, shortness of breath and reduced QoL. In this study, two items about pain were removed because they are covered by the BPI. The ESAS ranges from 0 = no influence to 10 = maximum influence [17] and has good validity and reliability for symptom assessment in cancer patients [19].

### Sample size

A total of 264 patients were included, 132 (66 from each hospital) in the control group and 132 (66 from each hospital) in the intervention group. Assuming an expected difference in pain intensity of 10% in the BPI, 65 individuals in each group are required to obtain 80% power at a significance level of 0.05; thus 130 patients from each hospital were necessary.

### Statistical methods

Statistical analyses were carried out with IBM SPSS Statistics 21 (IBM Corp., Armonk, NY, USA) using the Wilcoxon signed-rank, chi-square or McNemar test. Descriptive statistics were performed for all items in the BPI and ESAS. The responses from the control and the intervention group at baseline and follow-up were compared within each group, and outcome analyses were conducted. The baseline responses to the BPI items were compared between the control and the intervention group. Data from the medical records including documented pain and pain rating were analysed.

Analgesic prescriptions at baseline and follow-up for both groups were compared within each group. The completed BPI was analysed to identify nociceptive, neuropathic or mixed pain based on the patients’ descriptions of the pain characteristics [12, 13] and how they indicated painful areas on the body chart. Pain was categorized as either present at one or two pain sites or as multifocal (three or more locations). The responses from the control and the intervention group were compared.

### Ethical considerations

The patients were informed about the study orally and in writing. Participants provided both oral and written consent. All patients were informed that they would be contacted by phone to perform the follow-up assessment if they had been discharged from hospital, to which all consented orally. The study was approved by the Regional Ethics Board in Lund, Sweden (Nos. 2015/95, 2016/1066).

## Results

### Patient characteristics

The study included 155 women (58.7%) and 109 men (41.3%), median age 69 (range 18–92) years. The most common diagnoses were gastrointestinal cancer (25.8%), lung cancer (15.5%) and head and neck cancer (13.6%). Patient characteristics are presented in Table 1. Most patients had been diagnosed with cancer within the previous 3 years (71.6%). Among those with metastatic disease (non-curative), several were receiving ongoing chemotherapy or radiation therapy. Fifty-four (20.5%) were registered with a palliative team or an advanced home care team.

**Table 1:** Demographic and clinical characteristics of the participants at baseline (*n* = 264)

		*n*	%
Age, years	Median (range)	69 (18–92)	
Sex	Men Women	109 155	41.3 58.7
Cancer type	Gastrointestinal Lung cancer Head and neck Gynaecological Haematological Urological Breast cancer Other diagnosis	68 41 36 30 30 27 18 14	25.8 15.5 13.6 11.4 11.4 10.7 6.8 5.3
Type of treatment	Surgery^1^ Radiotherapy^1^ Radiotherapy^2^ Chemotherapy^3^	111 118 47 206	42.0 44.7 17.8 78.0
Treatment intention	Curative/adjuvant Non-curative	80 184	30.3 69.7

^1^More than 3 weeks ago; ^2^at present; ^3^at any time

### Flow chart of the study

**Fig 1:**
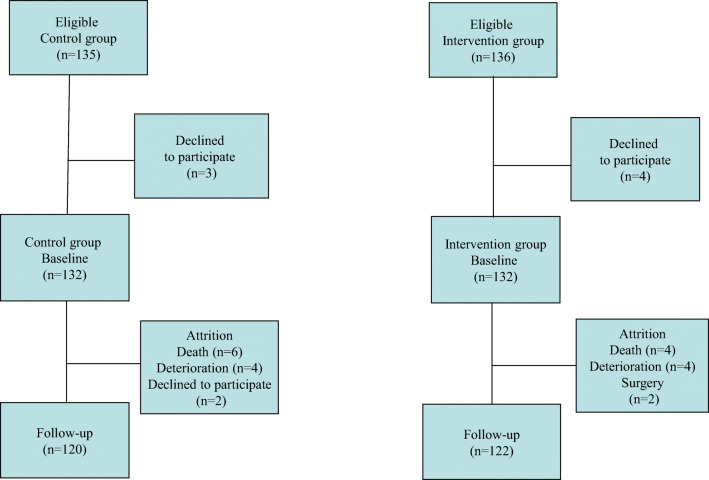
Flow chart of the study

Among eligible patients, a total of seven declined to participate. At baseline, 132 patients participated in the control group and 132 in the intervention group. Figure 1 presents a flow chart of the study including the attrition rate. A total of 120 patients from the control and 122 from the intervention group participated at both baseline and follow-up.

### Documentation and pain assessment

Notes in the medical records concerning pain were available for 187 (70.8%) of the 264 participants at baseline. Documented pain assessment over the past 24 h was available in the medical records for 81 patients (30.7%). Ninety-nine patients (37.7%) stated that they had used the pain assessment scale previously during their hospital stay.

### Patients’ perception of pain relief

**Fig. 2:**
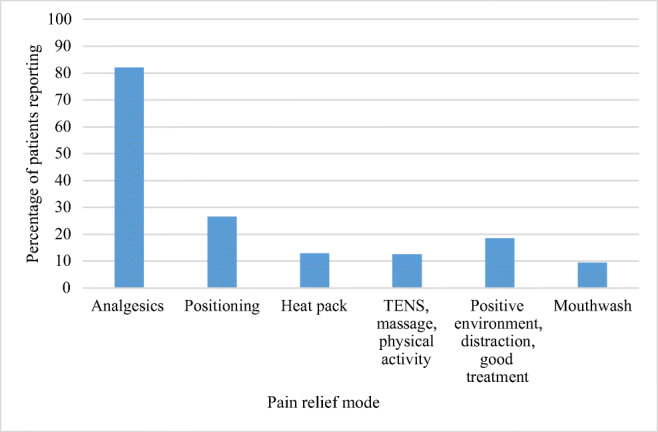
Patient-reported means of pain relief at baseline. *TENS* transcutaneous electrical nerve stimulation

At baseline (*n* = 264), 82% stated that analgesics provided pain relief and 26.5% reported that a change in position alleviated pain. Heat (12.9%), transcutaneous electrical nerve stimulation (TENS), massage and physical activity were also described as providing pain relief (12.5%). Furthermore, a positive environment, distraction and good treatment from the staff further provided relief (18.5%). Patients with ongoing radiation therapy for head and neck cancer stated that rinsing the mouth with water, saline solution or local anaesthesia (mouthwash) provided pain relief (9.4%) (Fig. 2). One patient reported that a support brace was a soothing complement to analgesics.

### Brief Pain Inventory scores at baseline and follow-up

Significant differences in all items on the BPI were found in the intervention group at follow-up compared with baseline (*p* < 0.001). In the control group, significant differences were found regarding worst pain over the past 24 h (*p* = 0.020) and average pain (*p* = 0.022) (Table 2). There were no significant differences between the two groups at baseline, with all patients included (*n* = 264).

**Table 2:** Brief Pain Inventory (BPI) scores at baseline and follow-up (*n* = 242)

	Control group (*n* = 120)				Intervention group (*n* = 122)			
Item	Baseline (mean)	Follow-up (mean)	*n*	*p* value*	Baseline (mean)	Follow-up (mean)	*n*	*P* value*
Worst pain	7.1	6.7	120	0.020	7.4	5.6	121	< 0.001
Least pain	2.6	2.7	120	0.488	2.7	2.1	120	< 0.001
Average pain	4.8	4.5	118	0.022	4.9	3.9	120	< 0.001
Current pain	3.9	3.9	120	0.609	4.0	3.2	122	< 0.001
Pain relief, %	70%	70%	112	0.457	70%	80%	118	< 0.001
Pain interference with:								
General activities	5.8	5.7	120	0.943	6.5	5.3	119	< 0.001
Mood	5.4	5.4	120	0.816	5.9	4.7	119	< 0.001
Walking ability	4.4	4.3	119	0.360	4.8	4.0	122	< 0.001
Normal work	5.9	6.3	89	0.137	6.4	5.7	88	< 0.001
Relations with other people	4.5	4.5	119	0.750	4.6	3.8	121	< 0.001
Sleep	4.2	4.0	120	0.212	4.4	3.3	122	< 0.001
Enjoyment of life	6.1	6.1	119	0.730	6.0	5.2	117	< 0.001

*Wilcoxon signed-rank test

### Pain characteristics

No significant differences in pain pathophysiology were seen between the control and the intervention group (*p* = 0.844); in all,153 patients (63%) experienced mixed pain, a combination of nociceptive and neuropathic pain, 34% reported only nociceptive pain and 3% experienced exclusively neuropathic pain. No significant difference was seen between the groups concerning pain site (*p* = 0.264); 60 patients (25%) had multifocal pain in three or more locations; 75% reported one or two locations. Several patients with ongoing radiation therapy had inflammatory components to their pain, such as erythema, ulcers, tenderness and swelling at the radiation site.

### Analgesic prescriptions

No significant differences regarding pain medication prescriptions were seen at follow-up in the control group. In the intervention group there was a significant increase in prescribed regular use of paracetamol (*p* = 0.008) at follow-up but a decrease in use of paracetamol as needed (*p* = 0.035), as well as a significant increase in strong opioids at a fixed schedule (*p* = 0.012). The addition of medications for neuropathic pain had increased at follow-up in the intervention group (*p* = 0.039) (Table 3). The dose of fixed-schedule opioids showed a significant increase in the intervention group (*p* = 0.021) at follow-up (not shown in the Table).

**Table 3:** Analgesic prescriptions at baseline and follow-up in the control group (*n* = 120) and the intervention group (*n* = 122)

	Control group			Intervention group		
	Baseline	Follow-up		Baseline	Follow-up	
	*n* (%)	*n* (%)	*p* value*	*n* (%)	*n* (%)	*p* value*
Paracetamol						
Fixed schedule	69 (57.5)	71 (59.2)	0.727	65 (53.3)	77 (63.1)	0.008
As needed	27 (22.5)	27 (22.7)	1.000	35 (28.7)	26 (21.3)	0.035
NSAIDs	14 (11.7)	12 (10.0)	0.688	8 (6.6)	12 (9.8)	0.125
Strong opioid po, iv, sc, TD						
Fixed schedule	76 (63.3)	80 (66.7)	0.219	89 (73.0)	98 (80.3)	0.012
As needed	93 (77.5)	92 (76.7)	0.100	108 (88.5)	110 (90.2)	0.500
Neuropathic pain drugs	15 (12.5)	16 (13.3)	1.000	23 (18.9)	30 (24.6)	0.039

*McNemar test, *iv* intravenously, *po* per os, *sc* subcutaneously, *TD* transdermally, *NSAID* non-steroidal anti-inflammatory drug

### Edmonton Symptom Assessment Scale scores at baseline and follow-up

No significant differences in assessed symptoms were seen at follow-up compared with baseline in the control group. In the intervention group, significant differences were found for all items except shortness of breath (Table 4).

**Table 4:** Edmonton Symptom Assessment Scale scores at baseline and follow-up (*n* = 242)

	Control group (*n* = 120)				Intervention group (*n* = 122)			
Item	Baseline (mean)	Follow-up (mean)	*n*	*p* value*	Baseline (mean)	Follow-up (mean)	*n*	*p* value*
Fatigue	6.0	5.9	120	0.892	6.0	5.5	121	0.002
Nausea	2.9	2.8	120	0.566	2.5	1.8	122	< 0.001
Sadness	4.1	3.9	119	0.374	4.1	3.6	121	0.001
Anxiety	3.6	3.4	119	0.251	4.0	3.7	122	0.004
Tiredness	5.7	5.4	119	0.204	5.3	4.8	122	0.001
Lack of appetite	5.6	5.6	120	0.675	5.7	4.8	122	< 0.001
Shortness of breath	3.4	3.5	120	0.480	3.2	3.0	122	0.404
Quality of life	6.0	6.0	120	0.861	5.9	5.3	117	< 0.001

*Wilcoxon signed-rank test

## Discussion

This study reports that pain intensity and other parameters affected by pain, in hospitalized patients with cancer decreased significantly, in an intervention using the BPI. Presenting patients’ completed questionnaires to their care team helped the healthcare professionals to focus on the patients’ pain, analyse the pain mechanism and adjust analgesic medications accordingly.

A large proportion of the patients in our study had undergone surgery, radiation therapy or chemotherapy. It is likely that in addition to tumour-related pain, some of the patients suffered from chronic pain following surgery, pain from radiation therapy or neuropathic pain due to chemotherapy. Pain is the most problematic symptom among patients undergoing radiation therapy for head and neck cancer [20]. Common diagnoses in our study included breast, prostate, renal and lung cancer, which are strong predictors of metastases in the vertebral body, ribs, hips, femur and tibia [21, 22], and 70% of the patients in our study had metastatic disease.

Pain was documented in the medical records of about 70% of the patients and pain assessment scores for 31%. Because all participants in this study had verified pain of moderate to severe intensity, this should have been documented, including a description of pain location, character and intensity according to the guidelines [12, 13]. Other studies report deficiencies in the documentation of pain reassessment by nurses [23], as well as a more comprehensive assessment completed [24]. Guidelines stipulate that continual pain assessment should be conducted and documented to ensure good pain control and achievement of treatment goals [12, 13]. Inadequate pain assessment and follow-up among our participants may have played a role in the failure to adequately address pain.

Many of the patients (82%) in our study reported that analgesics provided relief for their basic pain or breakthrough pain. The participants also used non-pharmacological methods to alleviate pain such as a changing position, heat, TENS, massage and physical activity. A friendly treatment, socializing with others, a pleasant environment, beautiful things and nature were sources of distraction and relief. Pain is a multidimensional experience [25], which might explain why patients stated that a friendly treatment and pleasant environment provided relief. Non-pharmacological interventions, patient education and improved referral and care coordination have emerged as important areas that require increased attention [26]. Treatment with complementary methods as part of multimodal pain management requires teamwork involving several professions [13]. Reports from the patients in our study confirm that a team approach to pain is important.

In the present study, significant differences between baseline (NRS 7.4) and follow-up (NRS 5.6) were seen in perceived worst pain over the past 24 h in the intervention group, while the negative impact of pain on other parameters also showed a significant decrease. The results of this study suggest an improvement for patients who were given the opportunity to communicate their pain to the care team through the BPI questionnaire; their pain was more adequately addressed, thereby providing a basis for initiating pain-relieving measures. Pain intensity ≥ 5 among cancer patients has been shown to significantly impact QoL by interfering with daily activities, mood, sleep, enjoyment and relationships [27].

Our findings are consistent with others showing that pain intensity can decrease when medications are adjusted by an experienced pain management physician [28], when pain management guidelines are followed [29] or when pain consultations are conducted each week by the radiation oncologist and anaesthetist [30]. In summary, a combination of several measures could considerably improve pain management for cancer-related pain and thereby alleviate unnecessary suffering. Pain in terminally ill cancer patients affects their sense of dignity [31]. Severe and unbearable pain causes cancer patients to despair, as it reduces bodily function and hope of improvement; patients want to be pain-free or receive adequate pain management [32].

In this study, 34% of patients had nociceptive pain, while 63% had mixed pain and 3% neuropathic pain only. Other studies have reported that neuropathic pain occurs in 44–67% of hospitalized cancer patients [33–35]. We and others have shown that neuropathic pain is common among cancer patients and that analysis of pain mechanisms is of vital importance to be able to alleviate pain. Neuropathic pain has been shown to affect daily activities [36]. The present study reveals that severe pain had a major impact on daily functions. Most patients with advanced-stage disease have at least two types of pain rooted in different aetiologies [37, 38], which could explain the high occurrence of mixed pain in our study.

At follow-up, we found a significant increase in regular use of paracetamol and strong opioids, in addition to medications for neuropathic pain and elevated doses of fixed-schedule opioids in the intervention group, which we interpret as one explanation for the significant decrease in pain levels. In the intervention group, 25% were prescribed analgesics for neuropathic pain at follow-up. This is a higher percentage than the 10% and 8% reported elsewhere [33, 36] but in line with Manfrida et al. [30], who report an increase of neuropathic pain drugs of up to 27% after their intervention. In our study, the completed patient body chart and description of pain characteristics in the BPI likely contributed to the increased prescription of medications to treat neuropathic pain. Furthermore, 80% of patients in the intervention group were prescribed opioids regularly at follow-up. The study by Manfrida et al. [30] likewise showed an increase in opioid use; the authors report that 85% of patients received a prescription for opioids after an intervention. In other studies that achieved lower pain levels, increased use of opioids [29] and opioid rotation [28] were the most common interventions.

We conclude that in many patients, improved pain management can be achieved through relatively simple adjustment of analgesics. It was further noticed that other administration methods, such as regional infusion, nerve block, intrathecal or epidural administration or intravenous/subcutaneous patient-controlled analgesia (PCA), could also have been tried in some of our patients with severe pain [5, 12, 13]. However, among these methods, only PCA was used in a few patients at the university hospital. There is a need to be more flexible regarding method of administration, type of medication and personalized treatment, in order to provide optimal pain relief in cancer patients.

The ESAS scores showed significant improvement in the intervention group for all measured parameters except shortness of breath. This can be interpreted as meaning that decreased pain has a beneficial effect on other symptoms in cancer patients. Of the symptoms assessed by the ESAS before the intervention, fatigue and QoL averaged about 6 on a scale of 0–10*,* other studies on cancer patients have also demonstrated a large influence of these items [34, 39, 40]. Patients with cancer often experience several disease and treatment-related symptoms concurrently, so-called symptom clusters [41]. These are assumed to have a synergistic effect on patient outcomes compared to single symptoms, e.g. the symptom cluster consisting of fatigue, pain, anxiety and depression has been found to impact QoL [42]. One study reported that neuropathic pain in cancer patients has a greater impact on physical and psychological symptoms assessed by the ESAS compared with nociceptive pain [34]. The majority of our participants suffered from neuropathic pain. The decrease of pain in the intervention group may have contributed to the significant improvement in ESAS scores at follow-up.

Limitations of this study are that there were differences in the time interval between baseline and follow-up, because the researchers were unable to follow up all participants at the same interval. Furthermore, some diagnostic groups were quite small; more participants within each diagnostic group could have provided additional information. Strengths in this study are that we used patient-reported data, that it is a relatively large sample and that the study was performed in two hospitals, of different characteristics, i.e. a university hospital and a county hospital.

## Conclusions

In conclusion, the present study provides valuable information about the effect of a multidimensional pain assessment intervention on cancer pain and other symptoms in hospitalized patients with cancer. The study demonstrates that use of the BPI can highlight and contribute to cancer pain relief and that decreased pain can have a positive effect on other pain-related dimensions and symptoms. This highlights the importance of healthcare staff treating pain in a professional way, identifying pain mechanisms, using guidelines and taking a team approach.

## References

[1] Haumann J, Joosten A, Van den Beuken-Van Everdingen MH (2017). Pain prevalence in cancer patients: status quo or opportunities for improvement?. Curr Opin Support Palliat Care.

[2] Van den Beuken-Van Everdingen MH, De Rijke JM, Kessels AG, Schouten HC, Van Kleef M, Patijn J (2007). Prevalence of pain in patients with cancer: a systematic review of the past 40 years. Ann Oncol.

[3] Van den Beuken-Van Everdingen MH, Hochstenbach LM, Joosten EA, Tjan-Heijnen VC, Janssen DJ (2016). Update on prevalence of pain in patients with cancer: systematic review and meta-analysis. J Pain Symptom Manag.

[4] IASP. 2008–2009 Global Year Against Cancer Pain 2008. Available at: www.iasp-pain.org/GlobalYear/CancerPain. Accessed August 2019

[5] Caraceni A, Hanks G, Kaasa S, Bennet MI, Brunelli C, Cherny N (2012). Use of opioid analgesics in treatment of cancer pain: evidence-based recommendations from the EAPC. Lancet Oncol.

[6] Kroenke K, Theobald D, Wu J, Loza JK, Carpenter JS, Wanzhu TU (2010). The association of depression and pain with health-related quality of life, disability, and health care use in cancer patients. J Pain Symptom Manag.

[7] Kwon JH (2014). Overcoming barriers in cancer pain management. J Clin Oncol.

[8] McGuire DB (2004). Occurrence of cancer pain. J Natl Cancer Inst Monogr.

[9] Kurita GP, Tange H, Farholt NM, Sonne AS, Strömgren L, Ankersen L (2013). Pain characteristics and management of inpatients admitted to a comprehensive cancer centre: a cross-sectional study. Acta Anaesthesiol Scand.

[10] Mercadante S, Portenoy RK (2016). Breakthrough cancer pain; twenty-five years of study. PAIN.

[11] Rayment C, Hjermstad MJ, Aass N, Kaasa S, Caraceni A, Strasser F (2013). Neuropathic cancer pain: prevalence, severity, analgesics and impact from the European Palliative Care Research Collaborative-Computerised Symptom Assessment study. Palliat Med.

[12] Ripamonti CI, Santini D, Maranzano E, Berti M, Rolia F, on behalf of the ESMO Guidelines Working Group (2012). Management of cancer pain: ESMO clinical practice guidelines. Ann Oncol.

[13] Swarm RA, Paice JA, Anghelescu DL, Are M, Bruce JY, Buga S, Chwistek M, Cleeland C, Craig D, Gafford E, Greenlee H, Hansen E, Kamal AH, Kamdar MM, LeGrand S, Mackey S, McDowell M, Moryl N, Nabell LM, Nesbit S, O'Connor N, Rabow MW, Rickerson E, Shatsky R, Sindt J, Urba SG, Youngwerth JM, Hammond LJ, Gurski LA, BCPS (2019). Adult Cancer Pain, Version 3.2019, Clinical practice guidelines in oncology. J Natl Compr Canc Netw.

[14] Bennet MI (2009). The Brief Pain Inventory: revealing the effect of cancer pain. Lancet Oncol.

[15] Cleeland CS, Ryan KM (1994). Pain assessment: global use of the Brief Pain Inventory. Ann Acad Med Singap.

[16] Cleeland CS, Nakamura Y, Mendoza TR, Edwards KR, Douglas J, Serlin RC (1996). Dimensions of the impact of cancer pain in a four country sample: new information from multidimensional scaling. Pain.

[17] Bruera E, Kuehn N, Miller MJ, Selmser P, Macmillan K (1991). The Edmonton Symptom Assessment System (ESAS): a simple method for the assessment of palliative care patients. J Palliat Care.

[18] Stanhope J (2016) Brief Pain Inventory review. Oxford University Press, on behalf of the Society of Occupational Medicine 66:496–97 10.1093/occmed/kqw041 27067913

[19] Chang VT, Hwang SS, Feurman M (2000). Validation of the Edmonton Symptom Assessment Scale. Cancer.

[20] Shishodia NP, Divakar DD, Al Kheraif AA, Ramakrishnaiah R, Patham AA, Parine NR (2015). End stage palliative care of head and neck cancer: a case study. Asian Pac J Cancer Prev.

[21] Coleman RE (2006). Clinical features of metastatic bone disease and risk of skeletal morbidity. Clin Cancer Res.

[22] Mantyh PW (2015). Bone cancer pain: from mechanism to therapy. Curr Opin Support Palliat Care.

[23] Song W, Eaton LH, Gordon DB, Hoyle C, Doorenbos AZ (2015). Evaluation of evidence-based nursing pain management practice. Pain Manag Nurs.

[24] Herr K, Titler M, Fine P, Sanders S, Cavanaugh J, Swegle J (2010). Assessing and treating pain in hospices: current state of evidence-based practices. J Pain Symptom Manag.

[25] McGurie DB (1992). Comprehensive and multidimensional assessment and measurement of pain. J Pain Symptom Manag.

[26] Lovell M, Agar M, Lucket T, Davidson PM, Green A, Clayton J (2013). Australian survey of current practice and guideline use in adult cancer pain assessment and management: perspectives of palliative care physicians. J Palliat Med.

[27] Serlin RC, Mendoza TR, Nakamura Y, Edwards KR, Cleeland CS (1995). When is cancer pain mild, moderate or severe? Grading pain severity by its interference with function. Pain.

[28] Manfredi PL, Chandler S, Pigazzi A, Payne R (2000). Outcome of cancer pain consultations. Cancer.

[29] Chang VT, Hwang SS, Kasimis B (2002). Longitudinal documentation of cancer pain management outcomes: a pilot study at a VA medical center. J Pain Symptom Manag.

[30] Manfrida S, Masiello V, Cellini F, Adducci E, Polidoro L, Longo S (2019). IMproved MAnagement (IM-MA study) in cancer-related pain: the value of a joint approach by an integrated team of radiotherapist and anesthetist. Support Care Cancer.

[31] Oechsle K, Wais MC, Vehling S, Bokemeyer C, Mehnert A (2014). Relationship between symptom burden, distress, and sense of dignity in terminally ill cancer patients. J Pain Symptom Manag.

[32] Pathmawathi S, Beng TS, Li LM, Rosli R, Sharwend S, Kavitha RR, Christopher BC (2015). Satisfaction with and perception of pain management among palliative patients with breakthrough pain: a qualitative study. Pain Manag Nurs.

[33] Roberto A, Deandrea S, Greco MT, Corli O, Negri E, Pizzuto M, Ruggeri F (2016). Prevalence of neuropathic pain in cancer patients: pooled estimates from a systematic review of published literature and results from a survey conducted in 50 Italian palliative care centres. J Pain Symptom Manag.

[34] Ulas S, Eyigor S, Caramat I (2018). Quality of life and neuropathic pain in hospitalized cancer patients: a comparative analysis of patients in palliative care wards versus those in general wards. Indian J Palliat Care.

[35] Lundorff L, Peuckmann V, Sjogren P (2008). Pain management of opioid-treated cancer patients in hospital settings in Denmark. Acta Anaesthesiol Scand.

[36] Oosterling A, Te Boveldt N, Verhagen C, Van der Graaf W, Van Ham M, Van der Drift M (2016). Neuropathic pain components in patients with cancer: prevalence, treatment, and interference with daily activities. Pain Pract.

[37] Higginson IJ, Murtagh F (2010) Cancer pain epidemiology. In: Burera E, Portney RK (eds) Cancer pain. Assessment and management, vol 3. Cambridge University Press, pp 37–52

[38] Koh M (2010) Cancer pain syndromes. In: Burera E, Portney RK (eds) Cancer pain. Assessment and management, vol 4. Cambridge University Press, pp 53–88

[39] Shin SH, Hui D, Chisholm GB, Kwon JH, San-Miquel T, Allo JA (2014). Characteristics and outcomes of patients admitted to the acute palliative care unit from the emergency center. J Pain Symptom Manag.

[40] Stromgren AS, Niemann CU, Tange UB, Farholt H, Sonne NM, Ankersen L (2014). Quality of life and symptoms in patients with malignant diseases admitted to a comprehensive cancer centre. Support Care Cancer.

[41] Kim HJ, McGuire DB, Tulman L, Barsevick AM (2005). Symptom clusters: concept analysis and clinical implications for cancer nursing. Cancer Nurs.

[42] So WK, Marsh G, Ling WM, Leung FY, Lo JC, Yeung M (2009). The symptom cluster of fatigue, pain, anxiety, and depression and the effect on the quality of life of women receiving treatment for breast cancer: a multicentre study. Oncol Nurs Forum.

